# 3D Functional Corneal Stromal Tissue Equivalent Based on Corneal Stromal Stem Cells and Multi-Layered Silk Film Architecture

**DOI:** 10.1371/journal.pone.0169504

**Published:** 2017-01-18

**Authors:** Chiara E. Ghezzi, Benedetto Marelli, Fiorenzo G. Omenetto, James L. Funderburgh, David L. Kaplan

**Affiliations:** 1 Department of Biomedical Engineering, Tufts University, Medford, Massachusetts, United States of America; 2 Department of Ophthalmology, University of Pittsburgh School of Medicine, Pittsburgh, Pennsylvania, United States of America; Oklahoma State University Center for Health Sciences, UNITED STATES

## Abstract

The worldwide need for human cornea equivalents continues to grow. Few clinical options are limited to allogenic and synthetic material replacements. We hypothesized that tissue engineered human cornea systems based on mechanically robust, patterned, porous, thin, optically clear silk protein films, in combination with human corneal stromal stem cells (hCSSCs), would generate 3D functional corneal stroma tissue equivalents, in comparison to previously developed 2D approaches. Silk film contact guidance was used to control the alignment and distribution of hCSSCs on RGD-treated single porous silk films, which were then stacked in an orthogonally, multi-layered architecture and cultured for 9 weeks. These systems were compared similar systems generated with human corneal fibroblasts (hCFs). Both cell types were viable and preferentially aligned along the biomaterial patterns for up to 9 weeks in culture. H&E histological sections showed that the systems seeded with the hCSSCs displayed ECM production throughout the entire thickness of the constructs. In addition, the ECM proteins tested positive for keratocyte-specific tissue markers, including keratan sulfate, lumican, and keratocan. The quantification of hCSSC gene expression of keratocyte-tissue markers, including keratocan, lumican, human aldehyde dehydrogenase 3A1 (ALDH3A1), prostaglandin D2 synthase (PTDGS), and pyruvate dehydrogenase kinase, isozyme 4 (PDK4), within the 3D tissue systems demonstrated upregulation when compared to 2D single silk films and to the systems generated with the hCFs. Furthermore, the production of ECM from the hCSSC seeded systems and subsequent remodeling of the initial matrix significantly improved cohesiveness and mechanical performance of the constructs, while maintaining transparency after 9 weeks.

## Introduction

There is a high demand for human cornea replacements with an estimated 10 million people worldwide suffering from corneal vision loss [1]. In the United States over 48,000 corneal transplants were performed in 2013 [2, 3]. The clinical answer to this increasing demand is currently narrowed to allogenic and synthetic materials [4]. In the case of corneal graft replacements, allogenic materials from human donors are the gold standard [2], but their applicability is restricted by quality-donor corneal graft availability, rejection, and a progressively growing failure rate [5–7]. Alternatively, synthetic keratoprostheses based on polymethylmethacrylate still necessitates corneal tissue donors, while also including complications such as corneal melting, uveitis, endophthamitis, and retinal detachment, limiting long-term success [2, 8–10]. Concurrent with the need for more corneal replacement tissues, the need to reduce animal testing for pharmaceutical and cosmetic products further stimulates the demand for human corneal tissue equivalents, to be used as *in vitro* preclinical tissue models. The necessity within the cosmetics industry to produce tissue analogs to replace animal testing continues to grow to replace more traditional eye irritation and toxicity tests [11].

In an effort to provide alternative corneal substitutes, the field of tissue engineering has pursued a variety of approaches, starting from purely cell-based systems [12–14], to decellularized tissues [15, 16] to the engineering of synthetic [17–19] and natural polymers [20–23] to sustain and direct cell growth and organization [24]. However, current results remain far from providing functional corneal tissue equivalents, able to resemble the structural, mechanical, and optical properties of the native tissue as well as its cellular populations [15]. In particular, the corneal stroma accounts for approximately 90% of the overall cornea thickness and is comprised of layers of aligned collagen fibrils, accountable for the mechanical resistance, which are then interspaced by proteoglycans [25]. These small proteoglycans, including decorin, lumican, keratocan, decorated with dermatan sulfate and keratan sulfate, are believed responsible in the maintenance of the interfibrillar spacing required for transparency, as well as contributing to the regulation of corneal hydration [26, 27]. Furthermore, the corneal stroma is populated by keratocytes. These cells are sandwiched between lamellae and maintain the matrix components of the lamellar connective tissue [28, 29]. Therefore, the highly organized collagen lamellae provide mechanical support and biophysical features required for transparency [30].

The majority of the approaches to engineer the corneal stroma are limited to two-dimensional (2D) cultures, where the substrate is optimized to sustain cell growth and organization on the surface of the material. In comparison, three-dimensional (3D] culture systems provide closer resemblance of native tissue architecture, and further guide cell organization and tissue development. In fact, cell adhesion molecules, associated with intracellular signalling and differentiation [31], can interact with the surrounding matrix in three dimensions, resembling the native spatial organization of integrin receptors in comparison to 2D substrates [32]. Therefore, the dimensionality of the culture environment strongly affects cellular organization and responses.

Among the natural polymers, silk protein in the form of films has been successfully used as a substrate to engineer *in vitro* the corneal epithelium and stroma layers [20, 21, 33–39], due to the optical and mechanical properties as well as its versatile processability [40]. In addition, silk films have been shown to be a suitable materials for corneal stroma reconstruction in a rabbit animal model [41]. Silk films can be further organized in a 3D multi-lamellar architecture for the growth of corneal stromal cells, by optimizing topography, surface chemistry, and the porosity of each layer [37]. In combination with a silk film, human corneal stromal stem cells (hCSSCs) derived from the limbus have shown the capacity to develop an *in vitro* 2D human corneal stromal tissue equivalent [20]. In fact, hCSSCs are capable of maintaining their keratocyte potential *in vitro* [42], responsible for the unique composition of the corneal stroma and ultimately for its mechanical and optical properties. Futhermore, these stem cells have been shown not to elicit an immune response, while under the same conditions corneal fibroblasts were immunogenic [43]. The data suggest that a bioengineered cornea populated with such immune privileged cells could provide a viable supplement to corneal replacements and in vitro models.

We hypothesize that the combination of engineered silk films and hCSSCs in a 3D multi-lamellar architecture will support the production of an optically and mechanically functional corneal stromal tissue equivalent in comparison to 2D previous approaches and to the culture of human corneal fibroblasts (hCFs), known to lose their keratocyte potential upon *in vitro* culture [44]. Specifically, hCFs have been extracted from human donor corneal stroma, while hCSSCs from human donor limbus. The goal of this work is to create a high fidelity tissue model of the corneal stroma that can be used to bridge the gap between clinical models and current 2D in vitro tissue models and be useful for diseased models.

## Materials and Methods

### Preparation of aqueous silk solution

Aqueous silk solution was prepared from *Bombyx mori* silkworm cocoons, following the experimental procedure described in our previous studies [45]. *B*. *mori* silk cocoons were purchased from Tajima Shoji Co. (Yokohama, Japan). Briefly, the cocoons were degummed by boiling in 0.02-M sodium carbonate (Sigma Aldrich, St Louis, MO) solution for 30 min. The extracted fibroin was then rinsed three times in Milli-Q water, dissolved in a 9.3-M LiBr solution yielding a 20% (w/v) solution, and subsequently dialyzed (MWCO 3,500) against distilled water for 2 days to obtain silk fibroin aqueous solution at the approximate concentration of 8% (w/v), as determined by gravimetrical analysis.

### Preparation of polydimethylsiloxane (PDMS) substrates

**Fig 1:**
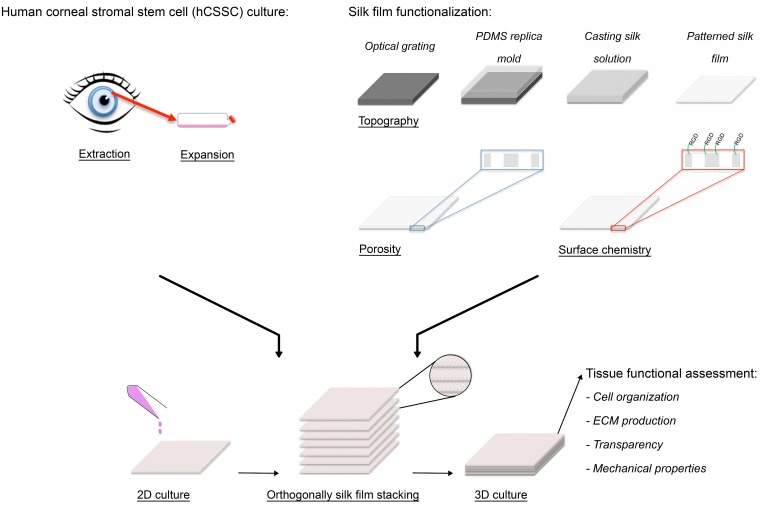
3D functional corneal stromal tissue equivalent preparation. Upon hCSSC extraction from human donor, cells were expanded in culture until seeding. Silk film topography, porosity and surface chemistry were optimized. hCSSCs were seeded on the silk films and cultured in 2D until confluent. Afterwards, 7 cellular silk layers were stacked in an orthogonal fashion to mimic the 3D physiological architecture. After 9 weeks in culture, the functional organization of the stroma equivalent was assessed based on cell organization, keratocyte-specific ECM production, optical and mechanical properties.

Sylgard 184 silicone elastomer kit (Fisher) was used in a 10:1 ratio and placed for 3 hours in a 70°C oven to cure. Patterned PDMS (Sylgard 184 Silicone Elastomer Kit, Dow Corning, Midland, MI) substrates were prepared by casting on reflective diffraction grating with grooves of 3.5 μm width and 500 nm depth (Edmund Optics, Inc, Barrington, NJ), as used in our previous study [37] (Fig 1). The PDMS substrates were washed in a 70% (v/v) ethanol solution and then rinsed in distilled water before casting the silk aqueous solution to generate the patterned silk films.

### Preparation of Arg-Gly-Asp-functionalized, patterned porous silk film

Silk films were prepared using PDMS replica mold over an optics glass substrate, as previously published [46] (Fig 1). Briefly, a mixture of 1% (w/v) silk fibroin and 0.05% (w/v) polyethylene oxide (PEO, MW ¼ 900,000; Sigma Aldrich, St. Louis, MO) solution was prepared to induce pore formation within the silk films. A 1.5 mL aliquot of 1% (w/v) silk solution was cast on patterned PDMS substrates of area equal to 45 x 45 mm^2^, resulting in films of 4 μm thickness after drying. Post-casting, the films were water-annealed in a vacuum oven (Isotemp Vacuum Oven Model 281A, Fisher Scientific, Pittsburgh, PA) with a water tray at 25°C, 20 mmHg vacuum for 60 min to induce beta-sheet in the protein structure [38]. Subsequently, silk films were maintained in distilled water under sterile condition for 3 days in order to leach out the PEO phase. The RGD surface functionalization of silk films was prepared as previously reported [37]. The films were presoaked in MES buffer (Pierce, Woburn, MA, 100 mM borate 150 mM and NaCl, pH 6.5) for 30 minutes. The—COOH groups from the aspartic and glutamic acid residues in the silk were activated by reaction with 1-ethyl-3- (dimethylaminopropyl) carbodiimide hydrochloride (EDC-HCl)/N-hydroxysuccinimide (NHS) solution (0.5 mg/mL of EDC and 0.7 mg/mL of NHS in MES buffer, pH 6.0) for 30 min at room temperature to form the stable and amine-reactive NHS-esters on the silk film surface. The activated silk films were then washed with MES buffer twice and subsequently treated in tripeptide Arg-Gly-Asp (RGD) solution (Bachen America Inc., Torrance, CA, 1 mg/mL of RGD in MES buffer, PH 6.0) at room temperature for 2 hours. The surface modified silk films were then washed in ddH_2_O two times. All silk films were sanitized with 70% EtOH for 30 minutes, and washed four times and store in phosphate-buffered saline (PBS) before use.

### Culture of human corneal stromal stem cells and human corneal fibroblasts

hCSSCs were isolated from collagenase digested limbal stromal tissue of human corneal rims from which central tissue had been removed for transplant. These were obtained from the Center for Organ Recovery & Education (Pittsburgh, PA), as previously described [42]. Cells at passage four were used for the experiments. As a comparison, P4 human corneal fibroblasts (hCFs) were kindly provided by May Griffith (Linköping University, Sweden). The cells were isolated from donor cornea. The cells were cultured in DMEM medium containing 10% FBS. The cultures were harvested with TrypLE^™^ (Life Technologies). hCSSCs were incubated with 1.0 mL of stem cell growth medium containing DMEM/MCDB-201 with 2% fetal bovine serum, 10 ng/mL epidermal growth factor, 10 ng/mL platelet-derived growth factor (PDGF-BB), 5 mg/ mL insulin, 5 mg/mL transferrin, 5 ng/mL selenous acid (ITS), lipid-rich bovine serum albumin (Albumax, Life Technologies, Grand Island, NY) 0.1 mM ascorbic acid-2- phosphate, 10_8 M dexamethasone, 100 IU/mL penicillin, 100 mg/mL streptomycin, 50 mg/mL gentamicin, 100 ng/ml cholera toxin [20]. Once confluent, hCSSCs were incubated in keratocyte differentiation medium (KDM) consisting of Advanced DMEM (Life Technologies) supplemented with 1.0 mM L ascorbic acid-2-phosphate (Sigma Aldrich, St Louis, MO), 2mM L-alanyl-L-glutamine (GlutaMax-1, Life Technologies), 50 mg/mL gentamicin (Life Technologies), 100 mg/ mL penicillin, 100 mg/mL streptomycin (Life Technologies), 10 ng/mL basic fibroblast growth factor (FGF-2, Sigma Aldrich) and 0.1 ng/mL transforming growth factor-beta3 (TGF-b3, SigmaAldrich) [20]. hCFs were cultured in high glucose DMEM containing 10% FBS, 100 mg/ mL penicillin, 100 mg/mL streptomycin (Life Technologies). The medium was changed twice per week for up to 9 weeks.

### hCSSC and hCF seeded multilayered silk film constructs preparation

hCSSCs and hCFs were seeded on single porous RGD-functionalized silk films at the seeding density of 5 x 10^4^ cells/cm^2^ and anchored at the bottom of a 100 mm petri dish with a silicone rubber o-ring. Cells were cultured on single film until 80% confluent and then sequentially stacked one by one, in order to have perpendicular alignment of the cells in between the layers (Fig 1). A total of 7 films were stacked per construct by absorbing the excess of media, then cut with surgical scissors in 10x10 mm^2^ samples and cultured in KDM into 24 well plates.

### Silk film morphological and structural characterization

In order to characterize silk film porosity, pore distribution within silk films was assessed with a Leica confocal laser scanning microscopy (CLSM) DMIRE2 (Wetzlar, Germany) with a 20X objective was used to take Z-stacks of the 4-μm films in bright field. Depth profiles and z-stack images were obtained by rendering two-dimensional images with 500 nm interval over 6 μm depth using Leica’s software.

In order to investigate the secondary structure of the silk films, Fourier transform infrared spectroscopy (FTIR) analysis was performed by multiple reflection, horizontal MIRacle attenuated total reflectance (ATR) (Pike Tech., Madison, WI) attached Jasco FT/IR-6200 spectrometer (JASCO, Tokyo, Japan). For each mid-infrared spectrum (4000–750 cm^-1^), 128 scans were collected in reflection mode at 4 cm^-1^ resolution. The Amide I band (1595–1705 cm^-1^) was used to investigate conformational differences in silk fibroin structure. The beta sheet content of silk films depends on the relative absorbance of the multiple resonances that compose the Amide I peak. In particular, the strong resonance band at 1610–1629 cm^-1^ is distinctive of beta sheet structure while the resonance band at 1640–1650 cm^-1^ is an indication of random-coil structure [47]. The fraction of beta sheet content was evaluated by Fourier self-deconvolution (FSD) of the infrared spectra covering the amide I region (1595–1705 cm^−1^) and curve fitting was performed by Opus 5.0 software [48, 49].

In order to characterize silk film topography, scanning electron microscopy (SEM) analysis was carried out on the porous patterned silk film. Dried samples were sputter coated with platinum/palladium (40 mA, 60 seconds) and imaged with a field emission SEM and 5 kV electron beam (Supra55VP, Zeiss, Oberkochen, Germany).

### Long term cell viability and distribution

Cell distribution as well as tissue construct morphology and extracellular matrix (ECM) production at 9 weeks in culture, were assessed by confocal laser scanning microscopy (CLSM) and histological analyses. For imaging with CLSM, cells were stained with calcein AM from LIVE/DEAD Viability/Cytotoxicity Kit (Life Technologies, Grand Island, NY) according to the manufacturer’s instructions. Briefly, cells were incubated for 60 min and then washed 3 times in PBS and imaged using a CLSM with excitation at 488 nm and emission at 499–537 nm. For histological preparation, samples (n = 3) were cut in 2 parts to study cell distribution and washed in phosphate buffered saline (PBS) and fixed in 10% neutral buffered formalin (Protocol, Fisher Scientific) overnight. Specimens were then processed, embedded in paraffin and cut in transverse sections of 7-μm thickness. Histological sections were then deparaffinised with xylene, rehydrated through a series of graded ethanol, and stained with hematoxylin and eosin (H&E). Histological sections were analysed with a light microscope (Leica DM500) using a 20X and 40X objectives.

### Whole-mount immunohistochemistry

The distribution of corneal-specific proteins within 3D hCSSC-seeded tissues in comparison to 2D hCSSCs and both 3D and 2D hCF constructs, was assessed with whole-mount immunohistochemistry against keratocan, keratan sulfate, lumican antibodies. Due to the structural changes imparted by the histological techniques, the silk constructs have been characterized only with whole mount immunohistochemistry and not on histological slides. At 9 weeks in culture, specimens were washed in PBS and fixed in 2.5% methanol-free paraformaldehyde (Polyscience Inc., USA) for 20 min at room temperature. Afterwards the specimens were washed three times in PBS, and stored at 4°C in PBS for further processing. To reduce nonspecific background staining, the samples were pre-incubated with PBS containing 2.5% horse serum for 20 min, rinsed in PBS and incubated in monoclonal primary antibodies diluted with 1% bovine serum albumin (Sigma-Aldrich) in PBS overnight at 4°C: keratocan (1:50, sc-33243 Santa Cruz Biotechnology Inc.), anti-keratan sulfate (1:50, sc-73518, Santa Cruz Biotechnology Inc.) Anti-lumican (1:50, ab168348, abcam). After washing the primary antibody, secondary antibodies Goat Anti-Mouse (1:250, ab7064, abcam), Goat Anti-Rabbit (1:250, ab50598, abcam), Donkey Anti-Goat (1:250, ab6881, abcam) were added to the samples and incubated for 1 h at room temperature. The stained samples were then maintained in PermaFluor Aqueous Mounting Medium (Thermo Fisher Scientific, Tewksbury, MA) until analysis.

### Reverse transcription and quantitative real-time polymerase chain reaction (qPCR)

hCSSC gene expression in comparison to hCF, expression levels of various genes were assessed by real-time quantitative Reverse Transcription—Polymerase Chain Reaction (RT-qPCR). hCSSCs and hCFs were cultured in 3D and 2D silk systems in 3 separate independent experiments. At 9 weeks, total RNA was extracted from hCSSC and hCF with RNeasy mini kit (Qiagen, Valencia, CA) and homogenized using QIAShredders (Qiagen, Valencia, CA) following manufacturer’s instructions. After RNA separation, nucleic acid concentration and integrity were determined with NanoDrop 2000 Spectrophotometer (Thermo Fisher Scientific, Tewksbury, MA). Total RNA (250 ng) was reverse-transcribed into complementary DNA (cDNA) with High-Capacity cDNA Reverse Transcription Kit ABI Biosystems (Life Technologies, Grand Island, NY) following manufacturer’s protocol. RT-qPCR was performed with a MX3000P (Stratagene, Agilent Technologies, Santa Clara, CA). Each PCR reaction contained 4 μl of cDNA, 1 μl of TaqMan^®^ Gene Expression Assay, 10 μl of TaqMan^®^ Gene Expression Master Mix (Life Technologies, Grand Island, NY) and 5 μl of RNase-free water. The TaqMan^®^ probes were keratocan (Hs00559942_m1), lumican (Hs00929860_m1), PDK4 (Hs00176875_m1), PTDGS (Hs00168748_m1), ALDH3A1 (Hs00964880_m1), ACTA2 (smooth muscle actin) (Hs00426835_g1), ribosomal RNA 18S (Hs99999901_s1). The cycling conditions were: 50°C for 2 min, initial denaturation at 95°C for 10 min, and 50 cycles of 15 seconds at 95°C and 1 min at 60°C. Relative quantification of target gene expression was achieved by normalizing against an endogenous reference gene (18S) to correct different amounts of input RNA, and then relating the expression of the target genes to a reference sample (cells at t = 0) using the -2ΔΔCt method [50].

### Mechanical assessment

The mechanical properties of 3D hCSSC-seeded constructs were characterized in comparison to as made (biomaterial alone, no cells) and hCF-seeded constructs, uniaxial tensile tests were performed (n = 3 per group) at 9 weeks with an Instron 3366 testing frame (Norwood, MA) equipped with a 10N capacity load cell and Biopulse pneumatic grip. Rectangular sample specimens (~6 x 30 mm^2^) were hydrated in PBS at 37°C before testing. The cross-sectional area of the samples was calculated by measuring the average thickness from histological measures. Samples were submerged in a temperature-controlled testing chamber (Biopuls) with PBS at 37°C. A displacement control mode with a crosshead displacement rate of 5 mm/s was used, and the gauge length was ~ 15–20 mm. The initial elastic modulus (EM), ultimate tensile strength (UTS) and % elongation to failure (ETF) were calculated from stress/strain plots. EM was calculated by using a least-squares (LS) fitting between 0.02 N load and 5% strain past this initial load point. UTS was determined as the highest stress value attained during the test and the ETF was the last data point before a >10% decrease in the load.

### Transparency measurements

Tissue construct transparency was also assessed, with transmission spectra of hCSSCs and hCFs seeded silk constructs characterized in comparison to as made constructs (biomaterial controls). The systems were measured by placing the samples in close proximity between two optical fiber probes (distance~5mm) coupled to a portable spectrometer (time constant: 5ms; average number: 10; wavelength: 350nm-1000nm, USB2000, Ocean Optics, Dunedin, FL).

### Statistical analysis

Data are expressed as mean ± standard deviation (SD). Data were analyzed for statistical significance by two-way ANOVA with a significance level p<0.05 and Tukey-Kramer and Holm-Bonferroni post-test methods for means comparison (Origin Pro v.8 software, OriginLab, USA).

## Results

### Silk film morphological and structural characterization

**Fig 2:**
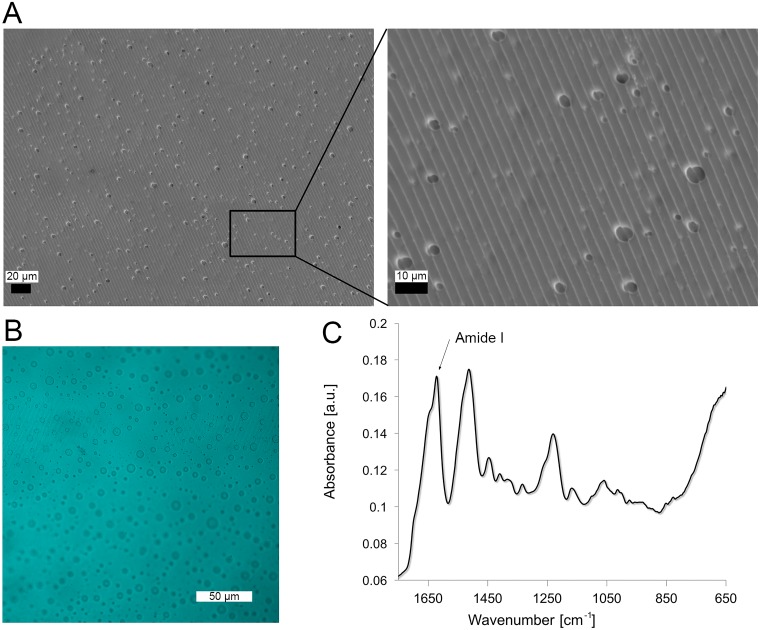
Morphological and structural characterization of silk film. A. SEM micrographs of the silk films, where high magnification images show the details of the surface pattern to guide cell alignment and porosity. B. Maximum intensity projection of CLSM analysis of silk films show 5 μm pores passing through the film. C. ATR-FTIR spectra of samples, where beta sheet crystalline content was induced upon water-annealing treatment. Samples were characterized by an Amide I absorbance split into two peaks centered at 1643 and 1621 cm^-1^.

After silk film preparation, PEO was leached out from the biomaterials in order to induce pore formation. SEMs showed homogenous pore distribution (Fig 2A) in the films. The method of fabrication based on PDMS replica mold over and optic glass substrate (Fig 1) conferred a regular pattern on the surfaces of the silk films, as shown in the high magnification micrograph (Fig 2A-insert). In particular, the groves were approximately 4 μm wide. Confocal scanning microscopy analysis confirmed 5 μm pores passing through the thickness of the film, as shown from the image profile in Fig 2B. ATR-FTIR analysis of the silk films showed the presence of beta sheet structure induced by the water annealing process (Fig 2C) [47). In particular, samples were characterized by an Amide I absorbance split into two peaks centered at 1643 and 1621 cm^-1^, indication of the presence of silk with amorphous and beta sheet structures, respectively. The beta sheet content of the protein was quantified by deconvoluting the Amide I spectra, as 35 ± 3%.

### Long term cell viability and distribution

**Fig 3:**
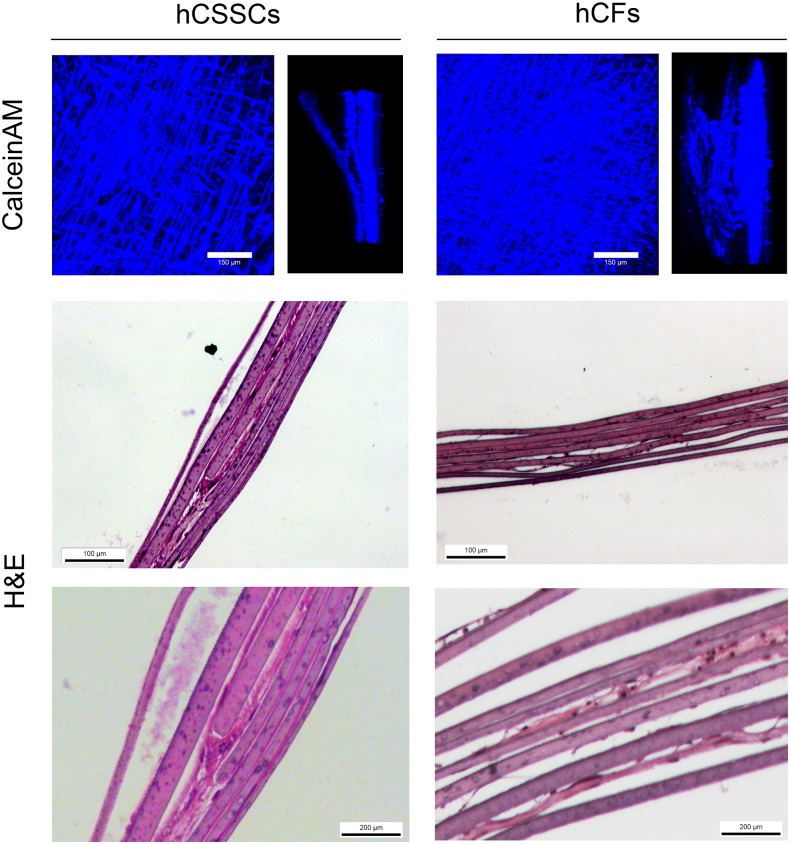
3D hCSSC and hCF tissue equivalent distribution at 9 weeks. Maximum intensity projections and side views of CLSM analysis of Calcein AM-stained hCSSCs and hCFs in the stroma equivalent. H&E staining of histological sections of stroma equivalent seeded with hCSSCs and hCFs showed evidence of ECM production at 9 weeks in culture. Scale bar = 100 and 200 μm.

To determine the long term cell viability and distribution within stroma tissue equivalent (STEq) for hCSSCs in comparison to hCFs(Fig 3), viability and cell distribution within the multilayered constructs were assessed through CLSM and histological sections after 9 weeks in culture. CLSM was used to monitor cell viability across the construct as well as cellular alignment, generating maximum intensity projections of calcein-AM fluorescence cells bound to the matrices (Fig 3). At 9 weeks in culture, hCSSCs and hCFs appeared aligned along the surface grooves of each biomaterial layer and distributed uniformly throughout multilayered thickness of both types of constructs, measured as average 150 μm, comprised of both silk films and ECM proteins. In agreement with the CLSM analysis, histological sections stained with H&E for both STEq displayed distribution throughout the construct thickness (Fig 3). Moreover, hCSSCs seeded STEq displayed significant amount of ECM production between the silk layers, while the hCF seeded STEq only had evidence of cell bodies between the layers.

### Whole-mount immunohistochemistry of corneal stroma-specific proteoglycans

**Fig 4:**
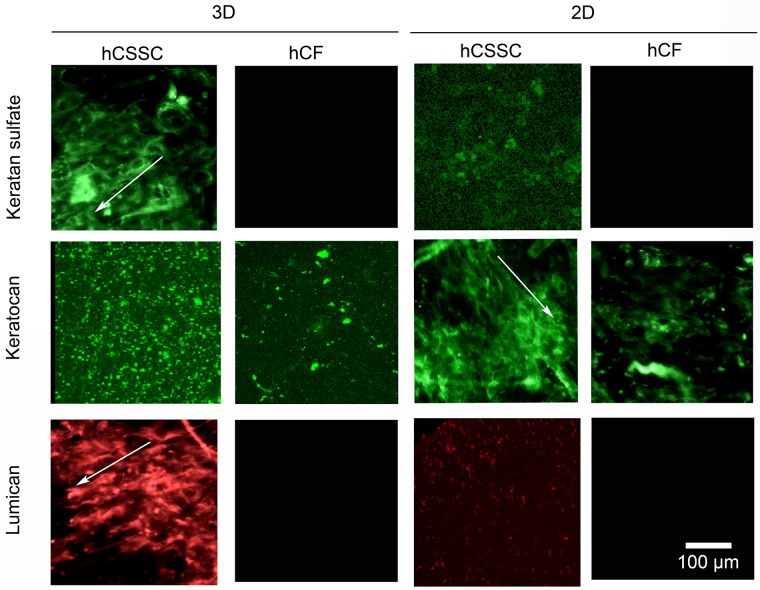
Immunofluorescent staining of corneal-specific proteins. 3D and 2D silk film constructs seeded with hCSSCs in comparison to hCFs were stained at 9 weeks in culture against keratocan, keratin sulfate, and lumican. The proteoglycans displayed preferential alignment along the silk film surface grooves (arrows). Scale bar = 100 μm.

The synthesis of corneal stroma-specific proteoglycans in the ECM was characterized by whole-mount immunohistochemical analysis (Fig 4). Keratan sulfate is the major glycosaminoglycan (GAG) present in the cornea stroma [51], while keratocan, and lumican are part of the family of the small proteoglycans which are modified with the keratan sulfate glycans. These represent key components of the corneal stromal tissue [52]. In particular, hCSSCs showed evidence of secretion of keratan sulfate as well as its proteoglycan protein cores in both the 3D and 2D cultures. Furthermore, the proteoglycans displayed preferential alignment along the silk film surface grooves (arrows in Fig 4). In contrast, the hCF fluorescence signal was weak and in some cases absent in both 2D and 3D systems.

### Changes in keratocyte gene expression

**Fig 5:**
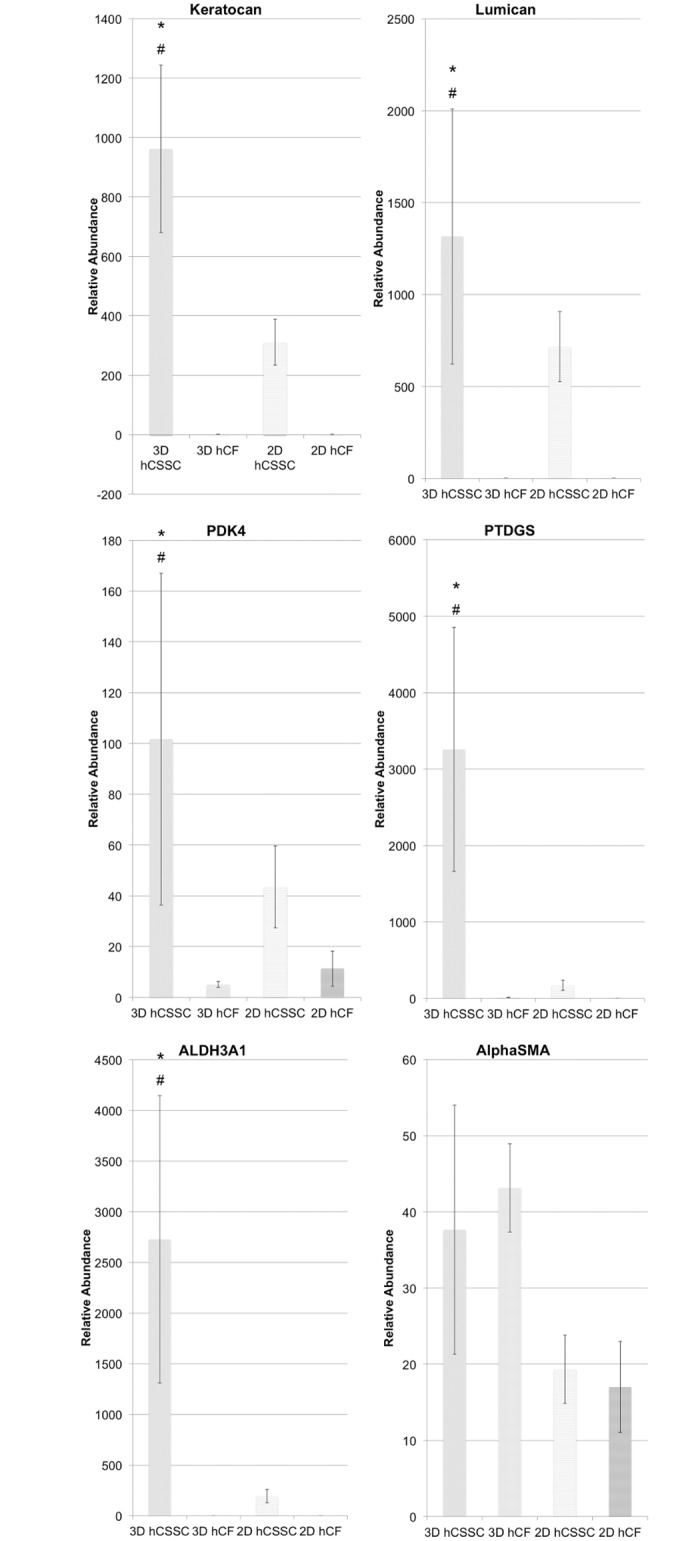
mRNA expression within hCSSC and hCF tissue equivalent in comparison to 2D culture. Changes in keratocyte gene expression within hCSSC and hCF in 3D culture at 9 weeks in comparison to culture on single silk films relative to hCSSC and hCF at day 0. At each time point, RNA was extracted and reverse-transcribed for RT-qPCR. RNA expression of each gene was first normalized against an endogenous reference gene (18S) and then related to the normalized expression level of the target gene at day 0 per each cell type. Keratocan, lumican, PDK4, PTDGS, and ALDH3A1 were significantly up-regulated for 3D hCSSC, suggesting the enhanced keratocytic phenotype in 3D cultures in comparison to 2D culture and hCF. * Significant effect of cell type (p<0.05); * significant effect of culture (p<0.05).

hCSSCs were switched to serum-free differentiation medium after reaching 90% confluence on the silk films. Gene expression of hCSSCs within the 3D STEq was analyzed by qPCR after 9 weeks in culture, in comparison to hCSSCs cultured in 2D single silk films and the hCFs maintained in both 2D and 3D environments. Fig 5 shows the gene expression profiles, normalized against 18S and relative to the initial gene expression at time 0 for both cell types. hCSSCs showed up-regulation in the relative gene abundance for characteristic gene markers for keratocytes, including keratocan, lumican, human aldehyde dehydrogenase 3A1 (ALDH3A1), prostaglandin D2 synthase (PTDGS), and pyruvate dehydrogenase kinase, isozyme 4 (PDK4). These markers were observed to be consistently higher in expression level when hCSSCs were cultured in 3D in comparison to 2D (p<0.05). hCFs showed no detectable expression of keratocan, lumican, PTDGS and ALDH3A1. Therefore, a significant effect of cell type on the marker expression was observed for all markers analyzed, except for alpha smooth muscle actin, where there was no significant effect of cell type or type of culture (p>0.05).

### Mechanical assessments

**Fig 6:**
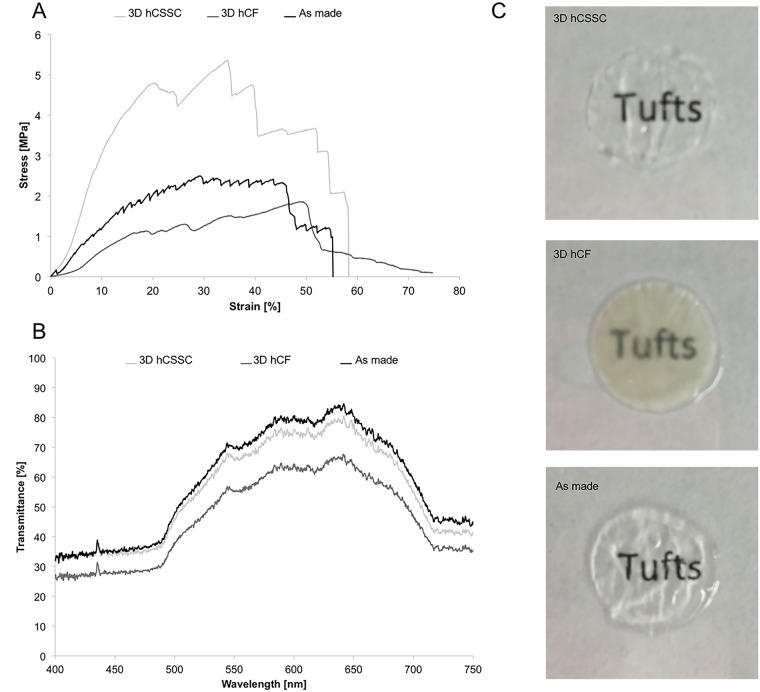
Tissue equivalent functional properties at 9 weeks in culture. A. Mechanical properties: Uniaxial tensile properties of hCSSC-seeded stroma equivalent in comparison to hCF-seeded and as made stroma equivalent. B. Optical properties: Transmittance measure of of hCSSC-seeded stroma equivalent in comparison to hCF-seeded and as made stroma equivalent. C. Macro images of each cornea equivalent are reported against written text to show differences in transparency.

STEq mechanical properties were characterized after 9 weeks in culture with uniaxial tensile testing to assess the effect of hCSSCs in comparison to hCFs on the mechanical performance of the systems. Representative stress-strain curves of hCSSC, hCF and as made STEq are reported in Fig 6A. The UTS and strain at failure were measured in the failure region, while the elastic modulus for each construct was calculated in initial linear region at low strain (Table 1). The tensile properties of hCSSC STEq demonstrated significantly higher elastic modulus, and UTS in comparison to the controls (p<0.05). Furthermore, the as made constructs (biomaterial alone, no cells) displayed higher elastic modulus and UTS in comparison to the hCFs-seeded construct at 9 weeks in culture (p<0.05).

**Table 1:** Elastic Modulus, UTS, and strain at UTS for hCSSC, hCF, and as made stroma equivalent.

Sample	Elastic Modulus [MPa]	UTS [MPa]	Strain at UTS [%]
hCSSC stroma equivalent	24.17 ± 5.83	4.66 ± 0.81	39.75 ± 11.46
hCF stroma equivalent	5.00 ± 2.04	1.68 ± 0.40	48.34 ± 16.10
As made stroma equivalent	10.11 ± 1.79	2.89 ± 0.49	37.12 ± 10.76

### Transparency

The measure of transmittance for hCSSCs STEq in comparison to hCFs and as made constructs was carried out after 9 weeks in culture (Fig 6B). Light transmittance properties in the visible range of hCSSCs construct were comparable to the as made sample. In contrast, optical properties of hCFs STEq were 25% lower in transmittance in comparison to hCSSCs STEq. Furthermore, macro images of each cornea equivalent are reported against written text to show differences in transparency (Fig 6C).

## Discussion

The main functions of the corneal tissue anticipate the design requirements of the biomaterial scaffolding material to support *in vitro* tissue growth and organization. In particular, corneal tissue needs to provide protection, transparency, and an adequate optical interface. Furthermore, the materials need to sustain the tissue biological functions, therefore supporting cellular growth and organization in 3D, while being implantable in the target site. Silk can be successfully used to answer corneal tissue needs, due to its mechanical and optical properties, its ability to support corneal cell growth and it is well tolerated when implanted intra-stromally [20, 37, 41]. The current biomaterial alternatives mainly rely on nano-fibrillar polyester, natural polymers blended polyethylene glycol and polylactic acid composites, as well as a variety of collagen-based constructs [18, 53–57]. These current materials and approaches are more limited in terms of optical properties, their ability to support keratocytic cellular responses or favorable *in vivo* behavior. We have previously shown the ability to generate 2D silk substrates able to support the growth and differentiation of cornea-derived cell populations [20, 21, 37]. In the present work, we aimed to investigate the behavior of hCSSCs in 3D sustained culture based on multilamellar silk film architectures in comparison to hCFs and to 2D culture conditions. Both cell types proliferated maintained viability throughout the entire construct thickness for the 9-week culture period. The homogenous distribution of the pores, as shown by CLSM, was accountable for the viable maintenance of the 3D tissue constructs for these prolonged cultures. Furthermore, both cell populations grew in alignment with the surface patterns present in each silk layer, in order to resemble the native organization within the tissue, where stromal cells are orderly aligned along the collagen fibrils, perpendicularly oriented depending on the stromal lamella [26].

hCFs have been previously used in combination with RGD-functionalized silk substrates, displaying their ability of growth and proliferation on such substrates, although lacking in keratocytic phenotype with a tendency towards a fibroblastic behavior [18, 37]. Upon culture in serum-free differentiation media, hCSSCs had previously differentiated into keratocyte-like cells with significant up-regulation of keratocyte gene markers and had deposited corneal stromal ECM, unlike hCFs, which had confirmed their tendency to differentiate into myofibroblast-type cells [20]. Endogenous ECM production after 9 weeks in culture was evident from H&E stained histological sections, where, particularly in the case of hCSSCs, matrix production was present between the silk layers of stroma equivalent, contributing to the structural cohesiveness of the tissue constructs. In contrast, hCF stroma equivalent did not show significant secretion of endogFenous matrix. Whole mount immunohistochemistry analysis of the ECM at 9 weeks showed the distribution of keratan sulfate and lumican, mainly within the hCSSC stroma equivalent, while evidence of keratocan was also evident within the hCF stroma equivalent. Generally, hCFs demonstrated a significant reduction in secreted ECM lacking corneal tissue-specific proteoglycan expression, such as keratan sulfate and lumican [52, 58]. Specifically, these molecules contribute to the maintenance of the interfibrillar spacing within the corneal lamellae, ultimately responsible for the tissue transparency. In comparison, hCSSC stroma equivalent was rich in native corneal tissue components, as previously shown only in 2D culture [20]. Particularly, the fluorescent signal was significantly greater when cultured within a 3D architecture, suggesting that the mimicking of the 3D native environment further supported the differentiation of hCSSC towards a keratocytic lineage. The study of keratocytic gene expression corroborated the differentiation state of hCSSC within the 3D culture environment in comparison to 2D and to hCFs.

Most cell populations would demand a 3D environment in order to arrange into a tissue-like structure under *in vitro* conditions. Cell adhesion receptors, distributed over the entire cell body interact with the surrounding matrix, significantly increasing the integrin receptor interaction density in comparison to 2D substrates, which do not resemble the cell arrangement in native tissues [31]. In this context, 3D architectures act at the solute diffusion level as well as at the protein binding level, therefore creating a tissue-scale solute concentration, as well as local intercellular gradients [32]. In the case of stroma equivalent, the positive effect of the 3D culture environment was extended to keratocyte-specific gene marker expression, including keratocan, lumican, PDK4, PTDGS, ALDH3A1, that were up-regulated in comparison to the relative cell type at time 0. These genes have been found highly expressed in differentiated keratocytes, and specifically when hCSSCs were cultured in serum- free medium supplemented with insulin and ascorbate; upregulated expression of these keratocyte-specific markers was reported [59]. Furthermore, comparable expression of alpha smooth muscle actin, a myofibroblast-specific gene marker, was observed in the case of hCSSCs and hCFs in both culture conditions.

Herein, we have demonstrated the ability of this multilamellar silk film structure to support the sustained 3D culture of hCSSCs into a functional *in vitro* tissue with mechanical properties comparable to the human tissue upon 9 weeks in culture [60]. In fact, hCSSCs in comparison to hCFs were able to improve the structural and mechanical performance of these cellularized constructs as shown by uniaxial tensile testing. The improved mechanical properties are determined by the endogenous keratocytic ECM production, demonstrated by histological analysis as well as immunostaining, that had determined increased cohesiveness between the silk layers, ultimately resulting in a functional mechanical performance. The impact of the endogenous ECM production on the optical properties of the multilamellar constructs was assessed by measuring the transmittance of hCSSC stroma equivalent after 9 weeks in culture, in comparison to as prepared sample and to hCF stroma equivalent at 9 weeks. However, the optical properties of hCSSC stroma equivalent were slightly reduced compared to the native cornea reported in the literature (85%) [61]. The difference against the literature could also be linked to the different hydration state of the two tissues and the measurement techniques. The resemblance of the ECM to native tissue, in both composition and architecture, was confirmed by the significant increase in optical transparency for the hCSSC stroma equivalent sample in comparison to hCF stroma equivalent, where keratocytic ECM proteins were not as strongly expressed based on immunohistochemistry and gene profiles analyses. Therefore, the composition of the endogenous ECM had a significant impact on the optical properties for the stroma equivalent, where fibrotic matrix production determined construct opacity over time. Based on the current results, future steps will be focused on investigating the interplay of human corneal epithelial cells and hCSSCs to further advance the complexity of this current tissue equivalent towards translational approaches.

## Conclusions

A 3D multi-lamellar silk film architecture was implemented to support the growth and differentiation of hCSSCs, in an effort to generate a functional corneal stroma equivalent, with optical and mechanical performances comparable to native cornea tissue. Endogenous ECM production within the hCSSC stroma equivalent showed similarities with native tissue in both expression and composition, resulting in improved optical and mechanical properties in comparison to hCFs over the 9-week culture period.
